# Supplementary material for: The Predatory Properties of Bradymonabacteria, the Representative of Facultative Prey-Dependent Predators
Source: Microorganisms. 2024 Oct 3;12(10):2008. doi: 10.3390/microorganisms12102008 (PMC11509652; doi:10.3390/microorganisms12102008)

Supplementary materials

The Predatory Properties of Bradymonabacteria, the Representative of Facultative Prey-Dependent Predators

Shuo Wang [1,2], Ya Gong [2], Guan-Jun Chen [2,3] and Zong-Jun Du [2,3,*]

[1] School of Life Science, Yantai University, Yantai 264005, China; wangshuomicro@ytu.edu.cn

[2] Marine College, Shandong University, Weihai 264209, China; gongya@sdu.edu.cn (Y.G.); guanjun@sdu.edu.cn (G.-J.C.)

[3] State Key Laboratory of Microbial Technology, Shandong University, Qingdao 266237, China

* Correspondence: duzongjun@sdu.edu.cn; Tel.: +86-631-5688303

Supplementary file 1 Primer and Taqman probe sequences

Supplementary file 2 Amplification programs

Supplementary file 3 Original data for absolute quantitative PCR

Supplementary file 4 Genes affiliated with T3SS or T3SS* encoded by strains of bradymonabacteria

Supplementary Fig. S1 Schematic of lethality tests of *Bradymonas sediminis* FA350^T to *Algriphagus marina* am2^T with 0.4 and 8.0 μm Transwells.

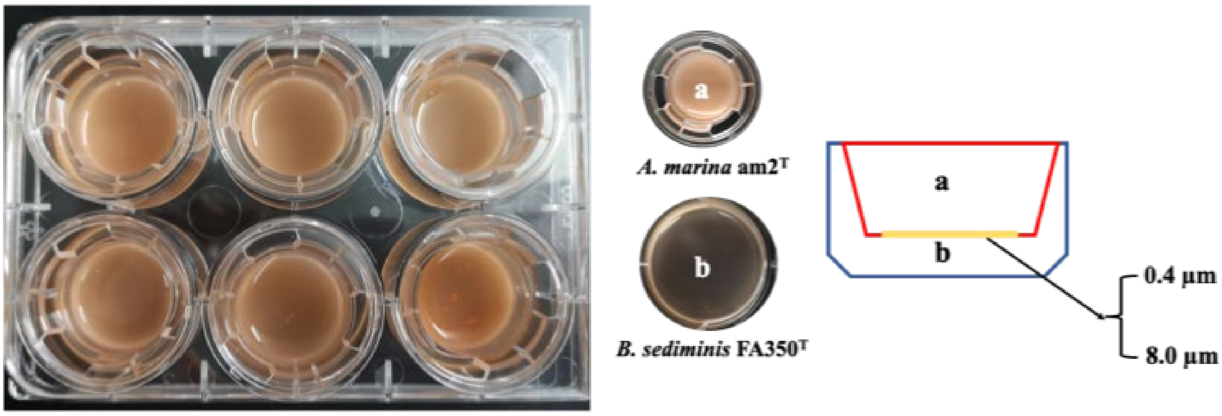

Supplementary Fig. S2 Standard curves of the specific gene concentration to the C_T value. A, Gene *flgM* representing *Bradymonas sediminis* FA350[T]; B, Gene *rubisco* representing *Algoriphagus marina* am2[T].

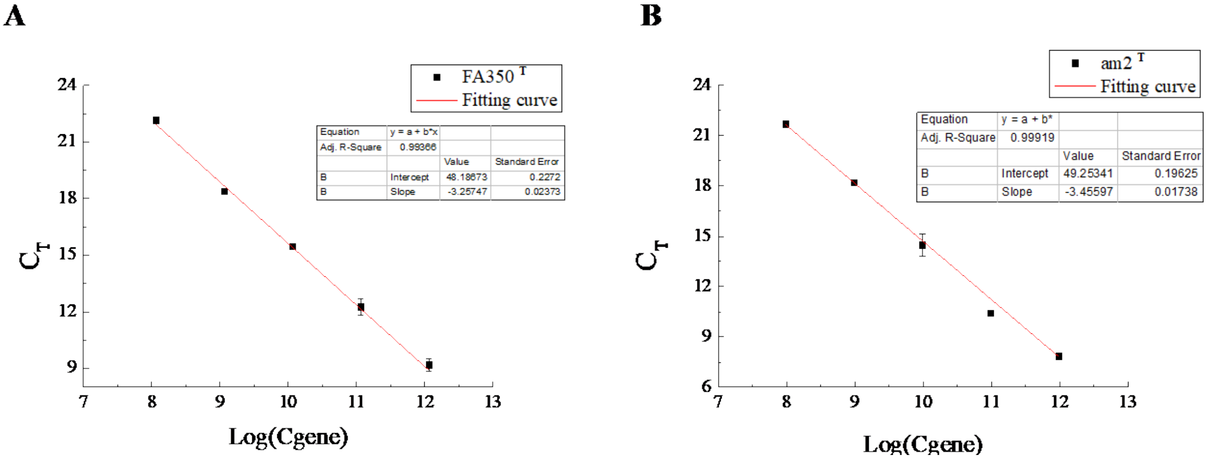

Supplementary Fig. S3 Facultative prey dependence of *Bradymonas sediminis* FA350^{T}. The plate of MA medium was spread with the suspension of *Br. sediminis*, then *Bacillus subtilis* and *Escherichia coli* were spot inoculated on the plate and co-cultured with the predator.

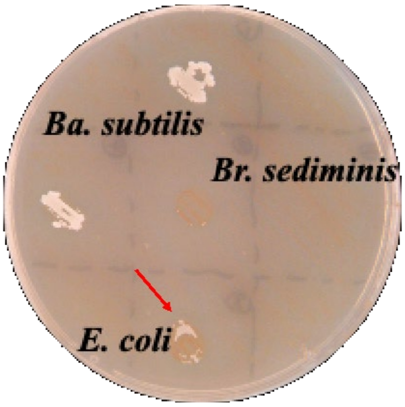

Supplementary Fig. S4 Growth curves of prey and predator and their corresponding standard curves of OD_{600} versus Log(CFU/mL).

A, *Algoriphagus marinus* am2^T; B, *Algoriphagus resistens* NH1^T; C, *Nocardioides gilvus* XZ17^T; D, *Nocardioides albus* JCM 3185^T; E, *Bacillus zeae* SDUM 602039; F, *Bacillus subtilis* 168; G, *Br. sediminis* FA350^T.

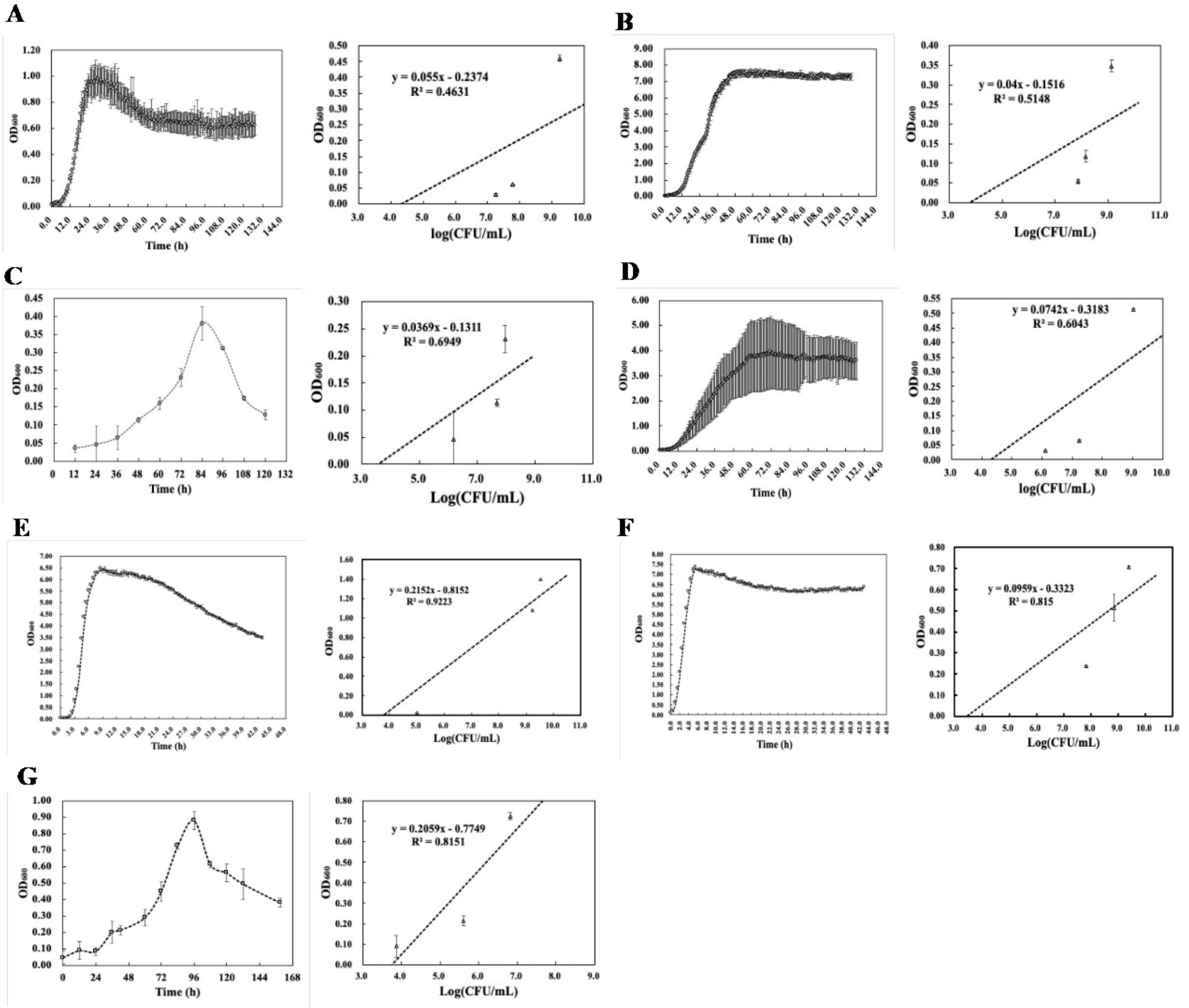

Supplement: Supplementary file 1 [file microorganisms-12-02008-s001.zip › Supplementary _files0922.pdf]
